# Supplementary material for: Extracellular-Vesicle-Associated UBE2NL and HIST2H3PS2 Promote Tumor Aggressiveness and Metastasis in Gynecologic Cancer
Source: Int J Mol Sci. 2025 May 18;26(10):4833. doi: 10.3390/ijms26104833 (PMC12112672; doi:10.3390/ijms26104833)
Supplement: Supplementary file 1 [file ijms-26-04833-s001.zip › Supplemental File S1.pdf]

Uncropped gel

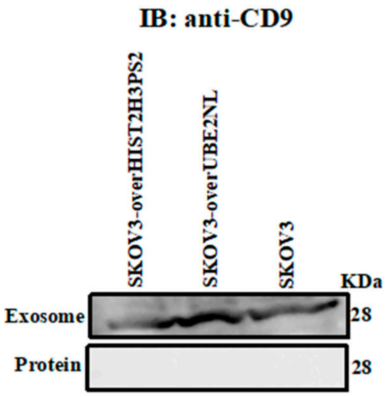


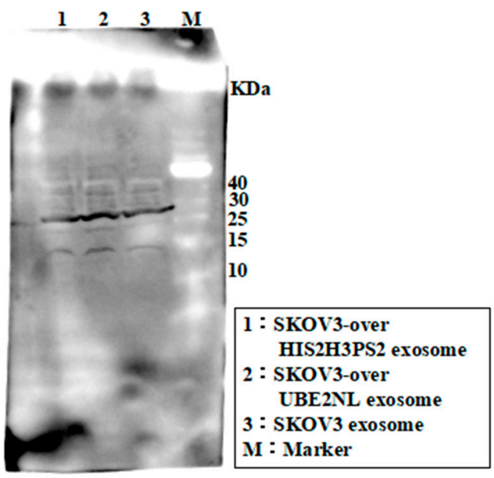


Uncropped gel

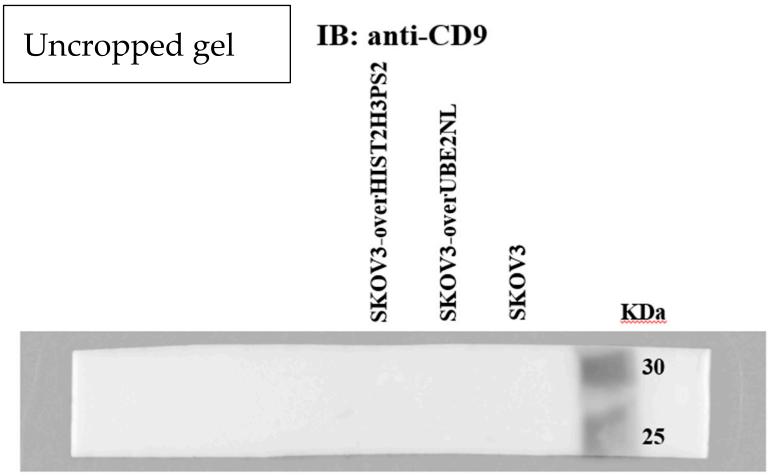


## Supplementary File S1 – TCGA Data Analysis Pipeline

TCGA Data Analysis Pipeline for Ovarian and Endometrial Cancer Transcriptome and Survival Study

### 1. Data Sources

Ovarian Cancer (OV):

- RNA-seq read count data (Level 3, serous type) for 372 samples were downloaded from the GDC Data Portal (https://portal.gdc.cancer.gov/).
- Gene annotation reference: GENCODE version 22 from GDC Reference Files

(https://gdc.cancer.gov/about-data/gdc-data-processing/gdc-reference-files).
- Clinical information: Pan-Cancer Atlas (https://gdc.cancer.gov/about-data/publications/pancanatlas).

Endometrial Cancer:

- Cohort created using GDC Data Portal (https://portal.gdc.cancer.gov/), resulting in 404 final samples after filtering.
- Clinical data included: stage, follow-up time, vital status, and recurrence.

## 2. Expression Normalization

TPM (Transcripts Per Million) values were calculated using the formula:

$$TPM_i = [(Read\ Count_i / Gene\ Length_i) / \sum_j (Read\ Count_j / Gene\ Length_j)] \times 10^6$$

## 3. Survival Analysis Workflow

### Ovarian Cancer

Software/Packages:
- R packages: survival, survMisc, survminer (version 0.4.9), ggplot2

Step-by-step Workflow:

1. Cox proportional-hazards model (coxph function) was applied to correlate TPM values with overall survival.
2. Hazard Ratio (HR) and Wald test p-values were calculated for each gene.
3. Genes were filtered with HR ≥ 2 and p-value ≤ 0.05.
4. For progression-free survival (PFS), optimal cut points were calculated using the cutp function (survMisc).
5. Samples were split into high and low expression groups based on TPM.
6. Kaplan-Meier plots were generated with ggsurvplot from survminer.

Sample Numbers Used:
- TCGA (serous type): 373 (OS), 177 (PFS)
- GEO + TCGA (all subtypes): 1656 (OS), 1435 (PFS)

### Endometrial Cancer

Expression Groups:

- Patients were divided into high and low expression groups of UBE2NL and HIST2H3PS2 using TPM cutoffs determined by survminer.

Survival Subgroups Analyzed:

- Early-stage (I-II) vs. advanced-stage (III-IV)
- Cancer stage (I, II, III, IV)
- Gene high vs. low expression

## 4. Summary of Tools Used

| Step | Tool/Package | Version |
| --- | --- | --- |
| TPM Calculation | Custom R script | – |
| Cox Regression | survival::coxph | 3.2-13 |
| Optimal Cut Point | survMisc::cutp | 0.5.5 |
| Kaplan-Meier Plot | survminer::ggsurvplot | 0.4.9 |
| Visualization | ggplot2 | 3.3.5 |

## 5. Notes

- All R scripts were run in R version 4.1.2 on Ubuntu 20.04 LTS.
- RNA-seq and microarray expression data were processed independently and normalized as required.
